# Prevalence and Factors Associated with Iron Deficiency and Anemia among Residents of Urban Areas of São Paulo, Brazil

**DOI:** 10.3390/nu13061888

**Published:** 2021-05-31

**Authors:** Cristiane Hermes Sales, Marcelo Macedo Rogero, Flávia Mori Sarti, Regina Mara Fisberg

**Affiliations:** 1 Department of Nutrition, School of Public Health, University of São Paulo, São Paulo 01246-904, Brazil; mmrogero@usp.br (M.M.R.); rfisberg@usp.br (R.M.F.); 2 School of Arts, Sciences and Humanities, University of São Paulo, São Paulo 03828-000, Brazil; flamori@usp.br

**Keywords:** anemia, iron deficiency, iron deficiency anemia, epidemiological surveys, iron overload, nutritional assessment, micronutrients, diet

## Abstract

Anemia is a worldwide concern. This cross-sectional population-based study examined the prevalence of iron-deficiency anemia (IDA) among residents of São Paulo (*n* = 898; 12–93 years), considering sociodemographic factors, dietary iron inadequacy, and food contributors to iron intake. Blood cell count and iron biomarkers were quantified. Dietary iron intake was measured using two 24-h dietary recalls. Iron intake inadequacy was estimated using a probabilistic approach. The prevalence of anemia was 6.7%, depleted iron stores 5.1%, and IDA 1.1%. Women of all age groups, older adults, and those who were underweight or obese had the highest prevalence of anemia, and female adolescents had the highest prevalence of depleted iron stores. Female adolescents and adults were more vulnerable to depleted iron stores. Male adults and older adults had a considerable prevalence of iron overload. Except for female adolescents and adults, all groups had mild probabilities of inadequate iron intake. The main food iron contributor was wheat flour. Hemoglobin concentrations were directly associated with being an adult, having a higher income, and inversely associated with being female. Serum ferritin concentrations were directly associated with age and inversely correlated with female sex. Residents of São Paulo had a low prevalence of anemia, iron deficiency, and IDA, and sociodemographic factors interfered with these parameters.

## 1. Introduction

Anemia is a global public health problem, affecting one-quarter of the world’s population, especially children, women of childbearing age, and older people [1,2,3,4,5,6]. Although anemia may be associated with genetic factors [4,5] or acquired conditions, such as hookworm infection, malaria [7], excess adiposity [5,8,9,10,11], and deficiency of several micronutrients [1,5,12], iron is highlighted among the micronutrients whose deficiencies can cause anemia [4].

It is estimated that 1.62 billion people worldwide have anemia [13]. Considering the historical prevalence of iron-deficiency anemia (IDA) worldwide [1,2,3,4,5], it is important to note that this condition is also observed in light of the double burden of malnutrition, in which both undernutrition and overnutrition can trigger IDA [9,14], as well as non-iron deficiency anemia [15]. Therefore, public policies on food and nutrition have been implemented to reduce the occurrence of this condition at the population level [3].

In Brazil, the typical process of nutritional transition with a double burden of malnutrition is currently ongoing, and following the global recommendations, a mandatory policy on the fortification of wheat and maize flours with iron has been effectively implemented since 2004. Given its safety and cost-effectiveness as a strategy to reduce IDA in the country [16], actions have been monitored by law [17] and by independent studies [18,19].

However, its efficacy has been controversial due to the diverse effects observed in individuals of different ages and sexes from different areas of Brazil [20,21,22,23]. In addition, it is difficult to evaluate the actual consumption of fortified foods [21,22], the adoption of low bioavailable iron additives [22], and the excess iron additive in some flours sampled throughout the country [18,19]. There is a lack of evidence on the proportion of individuals with dietary iron deficiency and on IDA prevalence in different population groups within large surveys that include specific results from blood samples. This may help differentiate IDA from other types of anemia and to support the appropriate interventions specific to the etiology of anemia, as pointed by Petry et al. [7] in a systematic analysis of national surveys.

Thus, this study aimed to estimate the prevalence of anemia associated with iron status, considering sociodemographic and lifestyle factors in a representative sample of residents from urban areas of the São Paulo city, and to evaluate the iron intake and the main food contributors to iron intake.

## 2. Materials and Methods

### 2.1. Study Population and Sample Design

Data were obtained from the 2015 Health Survey of São Paulo with a focus on nutrition (*Inquérito de Saúde de São Paulo com foco em Nutrição*; ISA-Nutrition 2015), a cross-sectional population-based survey [24,25].

The study population of ISA-Capital was delimited considering the 2010 Census urban situation of the census tracts. The strata were formed by the five geographical areas for health assistance in the São Paulo municipality—North, Midwest, Southeast, South, and East. In the sample planning, four domains were also considered: 1. male and female adolescents (12 to 19 years), 2. male adults (20 to 59 years), 3. female adults (20 to 59 years), and 4. male and female older adults (60 years or older). Twenty study domains were established considering geographic and demographic domains. Adjustments were made so that the health coordinators had equal data analysis potential, and it was considered a non-response rate of 40% and that 10% of households would be empty. Further details have been published elsewhere [25].

The individuals were randomly selected using a two-stage cluster sampling process: census tract and private households. In the first stage, 30 urban census tracts were randomly selected in each geographical area for health assistance in the São Paulo municipality. In the second stage, the households were selected in two distinct ways: for the sectors classified by the Brazilian Institute of Geography and Statistics as “common”, the households were systematically selected based on the list of households verified earlier in each area, and for the sectors classified as “special subnormal” that corresponds to sectors with favelas, the households’ segments were created considering an average size of six households [25].

The households of each geographical area were selected based on the selection made earlier in the areas with the rarest domain: adolescents in the Midwest and older adults in the other geographical areas. Based on these rarest domains, the number of the other domains were defined and selected. There was no selection within the household. All individuals belonging to the domain for which the household was selected were included [25]. For ISA-Nutrition, a subsample of 900 individuals, 150 for each age group and sex, was randomly selected from the sample of ISA-Capital, and to contemplate the five geographical areas and the inherent characteristics of each area [24].

During the first visit to 4059 individuals in their households, trained interviewers applied a structured questionnaire encompassing sociodemographic information, lifestyle information, self-reported morbidity and deficiencies, emotional health, quality of life, use of health services, preventive health examinations, immunizations, use of medicines, health-related behaviors, characteristics of the family and the household, and expenses with health. A subsample of 890 individuals answered the first 24-h dietary recall (24HR) during the first interview, followed by a second household visit of trained nurses to collect 12-h fasting venous blood samples, anthropometric measurements, and information about dietary supplements use. The subsample also answered a second 24HR by phone after 173 days (mean) of the second household visit [24].

Individuals who were heavy alcohol drinkers, those on enteral and/or parenteral nutrition, and pregnant or lactating women were not eligible to participate in ISA-Nutrition. During blood collection, individuals who had evidence of infectious disease had blood collection rescheduled to another date.

### 2.2. Sociodemographic Data, Lifestyle Information, and Anthropometric Measurements

Sex was classified as male or female. The age groups in the ISA-Nutrition survey were grouped into adolescents (12–19 years), adults (20–59 years), and older adults (60 years and older). The start age of adolescents was defined according to the Brazilian Statute of Children and Adolescents [26] which considers adolescents from 12 years old, and the others followed the World Health Organization’s [27] and the Brazilian Statute’s definitions of older adults [28]. The household heads’ education levels were defined as the number of years of education completed, and were categorized as <10 years (elementary school or less) or ≥10 years (high school or more). Family income *per capita* in international currency units, considering purchasing power parity, was divided into tertiles. Leisure-time physical activity was measured using the International Physical Activity Questionnaire [29]. Individuals who performed moderate-intensity exercise for at least 30 min per day, five days per week, or vigorous-intensity exercise for at least 20 min per day on three days per week were considered as individuals with sufficient physical activity; otherwise, they were considered as individuals with insufficient physical activity [30,31].

Weight and height were measured as described elsewhere [24], and the body mass index (BMI) was calculated as the quotient of weight (kg) over height squared (m^2^). BMI was classified as follows: for adolescents—underweight: <3rd percentile; healthy weight: >3rd percentile and <85th percentile; overweight not including obesity: >85th percentile and <97th percentile; obesity: >97th percentile [27]. For adults—underweight: <18.5 kg/m^2^; healthy weight: 18.5–24.9 kg/m^2^; overweight not including obesity: 25.0–29.9 kg/m^2^; obesity: ≥30 kg/m^2^ [32]. For older adults—underweight: <23.0 kg/m^2^; healthy weight: 23.0–27.9 kg/m^2^; overweight not including obesity: 28.0–29.9 kg/m^2^; obesity: ≥30 kg/m^2^ [33].

### 2.3. Iron Status and Anemia

Red blood cell counts were determined using an automated hematology analyzer (Sysmex XE-2100; Roche TOA Medical Electronics, Kobe, Japan). Serum ferritin concentrations were quantified using a chemiluminescent immunoassay (Ref. # 33020; Beckman Coulter Inc., Fullerton, CA, USA), serum iron status by colorimetric assay (Cobas; Roche Diagnostics GmbH, Mannheim, BW, Germany), and serum transferrin concentrations by kinetic nephelometry (Ref. # OSR6152; Beckman Coulter Inc., Fullerton, CA, USA). The transferrin saturation index (TS) was estimated using the ratio of serum iron (μg/dL) to the total iron-binding capacity multiplied by 100. The total iron-binding capacity was estimated using the ratio of transferrin (mg/dL) to 0.70 [34]. An enzyme-linked immunosorbent assay was used to quantify plasma high-sensitive C-reactive protein (usCRP) concentrations (Cat. # HEA821Hu; Cloud-Clone Corp., Houston, TX, USA). Additional details of the blood assessment methods are presented elsewhere [24].

Plasma usCRP concentrations were used as an indicator of acute-phase response proteins for infection/inflammation. Individuals with more than 2.0 mg/L [35] of us-PCR had serum ferritin concentrations adjusted by the regression correction approach, using linear regression with serum ferritin and plasma usCRP concentrations on a continuous scale [6,36]. usCRP values ≤ 2 mg/L were used to construct the following equation, where β represents the regression coefficient, that is, the variation in the *Y*-axis concerning the variation of one unit of the X variable:

Ferritin_adjusted_ = Ferritin_unadjusted_ − β × (usCRP_observed_ − usCRP_reference_)

Anemia was diagnosed based on hemoglobin concentrations of <13 g/dL for males aged ≥15 years or <12 g/dL for those in the other age groups [5]. Microcytosis and macrocytosis were identified using the mean corpuscular volumes of <80 fL and >100fL, respectively. Mean corpuscular hemoglobin <25 pg was an indicator of hypochromia and ≥25 pg indicated normochromia. Red cell distribution width >14% was an indicator of anisocytosis [37].

The reference levels for serum iron were 13–31 μmol/L (73–173 μg/dL) for males and 12–29 μmol/L (67–162 μg/dL) for females; for TS, it was 30–45% for both males and females, and that for transferrin was 215–365 mg/dL for males and 250–380 mg/dL for females [37].

Depleted iron stores were identified if serum ferritin was <15 μg/L [5], and severe risk of iron overload was indicated if it was >150 μg/L in females and >200 μg/L in males [3,5,6]. IDA was diagnosed when the individual concomitantly has anemia (hemoglobin concentrations of <13 g/dL for males aged ≥15 years or <12 g/dL for those in other groups) and depleted iron stores (serum ferritin was <15 μg/L) [5].

### 2.4. Dietary Iron Intake and Iron Supplement Use

Dietary data were collected using two 24HR applied on non-consecutive days using the Multiple Pass Method [38,39], and the Nutrition Data System for Research software (2014 version, Nutrition Coordinating Center, University of Minnesota, Minneapolis, MN, USA) was used to register the data obtained.

The nutritional values of foods included in the survey were compared with information available in the Brazilian national food composition table [40], and the Brazilian mandatory policy of maize and wheat flours’ iron fortification (4.2 mg Fe/100 g) [16], to allow for the correction of fortified foods’ nutritional values through a data correction routine developed using Stata software (14.0 version; StataCorp LP, College Station, TX, USA).

The usual iron intake of each individual was estimated using the multiple-source method [41]. The probability approach method was used to estimate the adequacy of dietary iron intake [42,43]. The percentiles of physiological iron requirements corresponding to women using hormonal contraceptives were considered, as reported by the Institute of Medicine [43].

During the application of the blood collection checklist, the individuals were asked if they were consuming any food supplements (vitamins, minerals, or other products). If so, the supplements were listed with the respective doses and forms of use. Among those identified, the ones with iron in their composition were identified to estimate the consumption of iron by supplements.

### 2.5. Foods and Beverages That Contribute to Iron Intake

Foods and beverages reported in the 24HR (*n* = 1002) included the frequency of intake and the corresponding amount of dietary iron consumed. Foods and beverages were grouped according to the What We Eat in America food classification system based on the National Health and Nutrition Examination Survey, designed by the Food Surveys Research Group of the United States Department of Agriculture [44]. The categorization follows the principle of grouping food and beverages considering the similarities identified in terms of culinary use and nutrient content, adapted to Latin American dietary habits [45].

The foods and beverages identified were distributed in the main food groups suggested by the United States Department of Agriculture: (1) dairy products, (2) protein foods, (3) mixed dishes, (4) grains, (5) snacks and sweets, (6) fruits, (7) vegetables, (8) non-alcoholic beverages, (9) alcoholic beverages, (10) water, (11) fats and oils, (12) condiments and sauces, and (13) sugars [44,45].

Considering the similarities of foods and beverages in terms of culinary use and nutrient content, each main group (*n* = 13) was subdivided into 40 subgroups, which were subsequently subdivided into 118 food and beverage categories to provide a greater level of detail of the 1002 foods and beverages identified in the 24HR.

After categorization, a relationship was established between the amount of iron from each category of food and beverage consumed, and the total dietary iron ingested in the diet. The categories were ranked to establish the main contributors to iron intake among residents of São Paulo.

### 2.6. Statistical Analysis

Statistical analyses were performed using the survey data commands available in Stata software, which considers sampling weights (probability weights), clustering, and stratification of the survey design [46]. Categorical data were expressed as percentages and a 95% confidence interval (95%CI), and continuous data as a median and interquartile range.

Prevalence was compared using survey-weighted chi-square tests, and medians using the Theil–Sen test for complexity sample studies [47] for the comparison of medians according to age groups, age groups and sex, self-reported race, household head education, family income *per capita*, leisure-time physical activity, and BMI classification. A 5% significance level was used for these tests.

Skewness and kurtosis test proposed by D’Agostino, Belanger, D’Agostino [48] with an adjustment made by Royston [49], and the Kolmogorov–Smirnov test were used to verify the normality of data. Serum ferritin concentration was transformed into a natural logarithm. The associations between hemoglobin or serum ferritin concentrations (dependent variable) and sociodemographic and lifestyle factors (independent variables) were assessed using the stepwise multiple linear regression method.

The final models were tested by residual analysis, and for inclusion, a 20% significance level was considered, whereas a 5% significance level was adopted for the other steps. The homoscedasticity test of the independent variables with iron intake was not successful.

## 3. Results

The prevalence of anemia was 6.7% in the urban population of São Paulo. A higher prevalence of anemia was observed among those aged ≥60 years, females, and individuals classified as underweight and obese. Although there were no observed differences in the prevalence of anemia, females had lower hemoglobin concentrations compared to males, and the hemoglobin concentration decreased according to the reduction of the tertile of family income *per capita* (Table 1). There were no differences in the prevalence of anemia and hemoglobin considering the following subdivisions: self-reported race, household head education, and leisure-time physical activity (data not shown).

Among those with anemia (*n* = 63), 16.4% (95%CI 7.7–31.5) were also diagnosed with microcytosis, hypochromia, and anisocytosis; 2.6% (95%CI, 0.3–17.0) with microcytosis and hypochromia; 5.4% (95%CI 1.2–21.5) with microcytosis and anisocytosis; 4.8% (95%CI 1.1–18.5) with microcytosis; and 19.2% (95%CI 10.4–32.6) with anisocytosis.

Assessing serum ferritin status, a low prevalence of depleted iron stores (5.1%) was observed in the entire sample. On the other hand, 18.8% of the sample had a severe risk of iron overload. A higher prevalence of depleted iron stores was observed among adolescents, mainly among females. In contrast, a higher prevalence of severe risk of iron overload was observed among males, mainly among adults and older adults.

Although no differences were observed in the prevalence of the other subdivisions according to sociodemographic and lifestyle factors, ferritin concentrations increased according to family income *per capita*, and it was lower in the healthy weight group compared to other BMI groups (Table 1). There were no differences in the prevalence of serum ferritin concentrations and iron store classification, considering the subdivisions: self-reported race, household head education, and leisure-time physical activity (data not shown).

Considering the assessment of serum iron, transferrin, and TS only two individuals had concomitant low serum iron, high transferrin, and low TS. Of these, none had depleted iron stores, and only one also had anemia.

When serum ferritin concentrations were combined with hemoglobin concentrations, only 1.1% had anemia and depleted iron stores, which is characteristic of IDA, 4.0% had depleted iron stores without anemia, 4.3% had only anemia, and 1.3% had anemia associated with severe risk of iron overload (Table 2). Considering the stratifications, differences were observed in the age groups and sex, leisure-time physical activity, and BMI classifications (Table 2).

In general, the probability of inadequate dietary iron intake was low (13.9%), but it was considerably higher in female adolescents and adults, who had lower consumption of iron compared to males in all age groups. Dietary iron intake and the probability of inadequate intake decrease with age. Self-reported race and family income *per capita* did not seem to interfere with iron intake; however, lower household head education level seemed to influence, as well as to do insufficient leisure-time physical activity. Being overweight and obesity seemed to be linked to lower iron intake, especially being overweight, which is related to a higher prevalence of inadequate dietary iron intake compared with the other groups (Table 3).

Only 29 individuals used supplements with iron in their composition. Except for one male older adult who ingested a supplement containing 120 mg of iron (supplement brand “*Combiron fólico*”), the largest amount consumed was at a dose of 50 mg of iron per day, with ferrous sulfate being the vehicle. In general, considering the low prevalence of iron supplement use, the intake amount had no impact on the distribution of the groups (Table 3).

The top 10 food contributors provided more than 60% of dietary iron intake, and the main food contributor of iron for all population groups investigated was wheat flour, particularly yeast bread. Beans, peas, and other legumes, and red meat were also important contributors to the overall iron intake. Minor differences were observed in other foods, such as chicken and soups (Table 4).

In the multiple linear regression analysis, hemoglobin concentrations were directly associated with being an adult and in the 3rd tertile of family income *per capita* and inversely associated with being female. Serum ferritin concentrations were directly associated with being an adult and an older adult, and inversely associated with being female (Table 5). Body mass index classification did not meet the criteria of *p*-value < 0.20, to be used in both models. Others did not pass the homoscedasticity test.

## 4. Discussion

Our results show that, in general, there was a mild prevalence of anemia, depletion of iron stores, and inadequate iron intake, and IDA does not seem to be a public health problem among residents of the urban areas of São Paulo. However, female adolescents and female adults showed a higher prevalence of anemia, depletion of iron stores, and inadequate dietary iron intake. Male adults and male older adults had an increased risk of iron overload compared to other population groups. Anemia was more prevalent in those on the weights’ extremes. Regarding iron intake, higher intakes were observed among individuals with higher education levels, sufficient physical activity levels, and healthy weights.

IDA has been highlighted as a global public health problem for decades [1,2,3,4,5], and public policies based on food fortification and iron supplementation for specific groups [3] have been implemented worldwide [3,5,16,23]. There are some controversies in the literature regarding the effectiveness of mandatory fortification whether iron fortification would be effective [21,22,50].

In a study conducted in São Paulo, Brazil, the authors showed that between 2003 to 2008 there was a reduction of approximately 60% in the prevalence of dietary iron inadequacy [20]. The prevalence presented by these authors in 2008 concerning dietary iron [20] were similar to the observed in the present study, considering age groups and sex, and varied from mild to moderate, female adolescents and female adults being the groups with a higher prevalence of dietary iron inadequacy.

Despite these mild to moderate probabilities of dietary iron inadequacy observed in the present study and by Vieira et al. [20], public policies implemented in Brazil [16,51] appear to have been successful in reducing IDA among residents of São Paulo. Since the IDA prevalence observed in the present study was 1.1% in the entire population, and 2.8% and 1.5%, if we consider female adolescents and female adults, respectively. Furthermore, the identification of wheat flour as the main food contributor to dietary iron reinforce the effectiveness of iron fortification, with contributions exceeding those of usual food sources such as red meat and beans, which are foods commonly consumed in the Brazilian dietary pattern [52].

Petry et al. [7] pointed out that there is a need to differentiate the types of anemia, and recommended that there is a need to individualize anemia-reduction strategies and programs because there is large heterogeneity in the proportion of those with IDA among countries [1,7]. Petry et al. [7] highlighted that the proportion of IDA is lower than that previously assumed in about 50% of the countries assessed by them, and showed that anemia varies from 9.1% in Vietnam to 76.3% in Sierra Leone, and IDA from 0.1% in Georgia to 21.2% in Liberia [7]. It is important to remember that these percentages of anemia can be influenced by the presence of diseases [53,54,55]. The same heterogeneity in the prevalence of anemia was observed in Brazil: some geographical regions presented a prevalence of anemia (9.9%) [56], and other regions had a higher prevalence, especially in more vulnerable groups, such as pregnant women, children, indigenous and low-income individuals [57,58,59,60].

Assessing the prevalence obtained in the present study according to the WHO criteria for the classification of public health significance [3,6], the residents of São Paulo exhibited a mild prevalence of anemia estimated from blood hemoglobin concentrations (mild: 5.0–19.9%). The same was observed in the stratified groups assessed, except for adolescents, male adolescents, male adults, healthy weight, and overweight without obesity, who exhibited a prevalence of anemia ≤4.9%; therefore, this should not be considered a public health problem.

Regarding serum ferritin concentrations, in the entire sample, a mild prevalence of depleted iron stores was also observed, concomitant with a mild risk of iron overload. In the stratification, adolescents, female adolescents, and female adults also had mildly depleted iron stores, while other groups had no problems with depleted iron stores. Conversely, it is important to mention the severe risk of iron overload observed in the study, which was more prevalent than iron depletion among adults, male older adults, 2nd and 3rd tertile of family income *per capita*, and those who were overweight and with obesity that presented moderate risk (moderate: 20%–39%), and male adults who presented severe risk (severe: ≥40%),

A higher prevalence of depleted iron stores among females, compared to males, has been observed worldwide [1]. It can be suggested that the higher prevalence of anemia in female individuals than in male individuals is due to lower consumption of fortified foods by female individuals, according to previous studies [20,61], reducing the effectiveness of iron fortification of flours in this population group. Our results support this hypothesis, considering that females consumed fewer flour-based products and other food sources of iron.

Nevertheless, although we cannot confirm, and the genetic, hormonal, and clinical issues need to be better investigated, the increased consumption of these fortified foods observed in males compared to females, over the years may be influencing the males to have this increased iron overload observed in the present study, as well as among the individuals who have hereditary hemochromatosis, as pointed out 10 years ago by Fisberg and Tosatti [62] and Martins [63].

Cançado and Chiattone [64] showed that the frequency of the HFE gene in chromosome 6 with C282Y mutation among residents of São Paulo could explain this iron overload observed, which is three to eight times lower than the rate observed in whites from northern Europe. It is possible that in the present sample assessed there are other non-HFE-associated hereditary hemochromatosis genes or other genes involved in the iron metabolism that could explain the severe risk of iron overload in the present study, but at this moment we cannot evaluate. We have the intention to do this in the future.

On the other hand, it can be hypothesized that the lower consumption of fortified foods by female individuals, combined with the menstrual blood loss common in women of childbearing age, may be a possible protective factor against iron overload, even when they become older adults. Genetic factors associated with anemia could also be involved and need to be studied.

As depleted iron stores cannot be neglected in these women of childbearing age, the severe risk of iron overload among males is also a concern, considering the possibility of organ damage due to the pro-oxidant potential [43]. High iron concentrations in the body, particularly in the free form, have been associated with a higher risk of prevalent diseases in the population, such as cardiovascular diseases, diabetes [43,65], and hepatocellular cancer [43], leading to worse health outcomes [66,67,68,69,70,71,72,73]. Excessive intake of micronutrients also increases the risk of mortality [65] due to the increase in the production of reactive oxygen species and inflammation, as well as cellular apoptosis [43,65].

Ferritin is a ubiquitous and conserved iron storage protein that plays an important role in maintaining iron homeostasis, as well as serum ferritin, which is considered an acute phase reactant. In this sense, serum ferritin concentration may represent a better biomarker of inflammation than of iron status in the presence of inflammatory diseases [74]. In this context, some metabolic diseases, such as obesity and type 2 diabetes, which are considered chronic low-grade subclinical inflammatory diseases, are associated with higher serum ferritin [75,76,77,78].

It is widely discussed that anemia may be associated with a condition called anemia of chronic disease [11,55,79], which has a concomitant low serum iron, low iron-binding capacity, and normal or elevated serum ferritin, and this is properly included in the context of the nutritional transition [35]. It should be noted that the pathophysiology of anemia in chronic diseases is related to mechanisms underlying iron sequestration. The prevalence of anemia increases with advancing age [80], and overall or central adiposity and oxidative stress seem to act as a relevant cause of older adults chronic inflammatory condition [81]. This fact may explain, in part, the results observed concerning the higher prevalence of anemia in older adults.

Furthermore, the role of other micronutrient deficiencies, such as vitamins A, B2, B6, B9, B12, C, D, and E, zinc, and copper, which are known to also lead to anemia and changes in iron metabolism [1,5,12], should be investigated. Due to the presence of other metabolic conditions, such as diabetes, obesity, and gastrointestinal diseases [2,5,82], to allow for a better understanding of other types of anemia not associated with depleted iron stores in older adults.

It is interesting to observe that a healthy lifestyle, characterized in this study by physical activity performance and healthy weight, had a positive influence on lowering the occurrence of anemia and in improving iron stores, while weight extremes (obesity and being underweight) were associated with a greater prevalence of anemia. The higher occurrence of anemia in individuals who were underweight can be explained by dietary inadequacy compromising iron intake; however, among individuals with obesity, dietary inadequacy and other processes related to inflammation could be the main problem.

Moreover, our results indicate that individuals with more education, sufficient physical activity, and healthy weight have a better dietary iron intake, which can be related to more food awareness, while income did not interfere with dietary iron intake, as observed in Costa Rica [83], possibly because iron-fortified foods in Brazil are relatively cheap.

The probability of dietary iron inadequacies observed specifically in female adolescents and adults, indicated by the higher prevalence of depleted iron storages (associated or not with anemia), reinforces the need for policies directed at women of childbearing age worldwide [1,2,5,84].

An important recommendation for public policy refers to the focus on the promotion of vegetable sources of iron for women of childbearing age [43]. Considering meat consumption, especially red meat which provides more bioavailable heme-iron, its promotion must be judicious even among individual women of childbearing age since residents of São Paulo have characteristically large intakes of red meat [85]. Promoting only the consumption of iron-fortified foods such as wheat or maize flours may not be recommended because the consumption of these foods in large amounts may increase the risk of becoming overweight and associated conditions. Otherwise, it would be interesting to individually identify women with any health condition creating vulnerabilities to iron deficiency for treatment. It is important to develop additional strategies focused on women at risk of depleted iron stores, such as iron supplementation [86], especially considering that other supplementation and fortification strategies without specific targeting may increase the risk of iron overload, which was identified in male adults and older adults.

Therefore, our results also highlight the importance of establishing the maximum limits of iron addition in wheat and maize flour fortification, to minimize the risk of iron overload. Kira et al. [19] pointed out that wheat flour produced and commercialized in the São Paulo city had wide variations in iron content, and some flours included the amount of iron high enough to produce toxic effects. The maximum limit of 9.0 mg of iron per 100 g of flour was established in Brazil in 2017 to avoid this adverse risk [51].

However, it is important to recognize that this is a study based on cross-sectional data, and causal inference cannot be made, providing evidence on population characteristics for identification of risk groups, and supporting health strategies to tackle anemia as a whole. Potential limitations of the study design include the selection, classification, and confounding biases, which may be minimized by using appropriate statistical strategies, such as control variables, which were adopted in the present analysis.

Although the sample size may seem small, it was calculated to be representative from São Paulo, based on the most recent epidemiological data available, and considering the population demography in São Paulo city, to generate accurate estimates both at the municipal and regional levels, as described elsewhere [25].

Furthermore, the limitations regarding potential errors in the estimation of dietary intake due to the adoption of retrospective records of food intake may be recognized; however, it is important to highlight that data collection and processing of two non-consecutive 24HR were carefully planned and applied by trained interviewers, including correction of food composition to account for iron intake considering food fortification parameters applicable in Brazil.

## 5. Conclusions

There was a low prevalence of iron deficiency, anemia, and IDA among residents of the urban areas of São Paulo. Women of childbearing age comprised the population group with high susceptibility to depleted iron stores and presented a greater probability of dietary iron inadequacy, while male individuals were more susceptible to the severe risk of iron overload and had practically no dietary iron inadequacy. BMI, educational level, and physical activity had distinct influences on anemia, iron deficiency, and iron intake. The main foods contributing to iron intake in the study were foods with wheat flour in their composition, as well as beans and red meat, signaling the role of iron fortification programs for the Brazilian population. Future studies should focus on the risk of iron overload in certain population groups, analyzing the potential effects of diseases and genetic issues that may influence this context, in addition to factors related to nutritional transition and geographical and lifestyle factors.

## Figures and Tables

**Table 1:** Anemia and iron stores among residents of the urban area of São Paulo, according to sociodemographic and lifestyle factors: 2015 Health Survey of São Paulo.

Characteristics	Hemoglobin (g/dL)				Anemia *			Serum Ferritin (μg/L) †				Iron Stores Classification ‡				
	*n*	Median	Interquartile Range	*p*-Value ‖	%	95%CI	*p*-Value §	*n*	Median	Interquartile Range	*p*-Value ‖	Depleted Iron Stores		Severe Risk of Iron Overload		*p*-Value §
												%	95%CI	%	95%CI	
Entire sample	897	14.8	13.6–16.0	-	6.7	5.0–8.7	-	899	74.2	37.1–147.5	-	5.1	3.7–6.9	18.8	15.9–22.2	-
Age groups																
Adolescents; 12–19 y	290	14.4	13.5–15.6	0.243	3.8	2.2–6.7	0.028	289	37.6	23.5–60.7	<0.001	9.0	6.0–13.2	2.2	1.0–4.7	<0.001
Adults; 20–59 y	302	15.0	13.8–16.2		6.2	3.9–9.9		302	97.3	45.9–182.7		4.6	2.7–7.8	26.9	21.7–32.7	
Older adults; ≥60 y	305	14.7	13.6–16.0		10.2	7.1–14.3		308	103.0	57.5–163.9		1.9	0.8–4.3	18.6	14.3–23.8	
Age groups and sex																
Male adolescents	140	15.1	14.3–16.1	<0.001	0.8	0.1–5.4	0.015	140	48.3	34.9–72.8	<0.001	1.3	0.3–5.3	4.6	2.2–9.5	<0.001
Female adolescents	150	13.8	13.0–14.7		6.7	3.7–11.9		149	29.0	16.5–44.4		16.2	10.8–23.7	NO		
Male adults	153	15.7	14.8–16.4	<0.001	4.4	2.0–9.6	0.198	153	150.1	100.7–245.3	<0.001	NO		50.6	42.2–59.0	<0.001
Female adults	149	14.0	12.9–15.0		8.4	4.7–14.6		149	43.1	26.7–83.2		9.9	5.8–16.2	NO		
Male older adults	151	15.1	14.0–16.5	<0.001	9.4	5.5–15.6	0.665	152	134.1	67.4–190.9	0.001	2.0	0.6–6.1	35.8	28.0–44.3	<0.001
Female older adults	154	14.0	12.8–15.3		10.9	6.8–17.1		156	88.2	51.9–143.3		1.8	0.6–5.6	NO		
Family income *per capita* ¶																
1st tertile	269	14.4	13.4–15.9	0.002	7.0	4.2–11.5	0.783	269	61.5	33.2–120.2	<0.001	7.7	4.8–12.0	14.6	10.1–20.7	0.182
2nd tertile	244	14.7	13.6–16.2		6.1	3.5–10.5		245	80.7	34.2–152.0		5.7	3.1–10.2	20.1	14.8–26.6	
3rd tertile	240	15.0	14.0–16.4		5.3	2.9–9.6		241	97.3	44.4–159.9		3.0	1.4–6.5	22.1	16.3–29.4	
Body mass index classification **																
Underweight	70	14.8	13.4–15.7	0.902	10.4	5.0–20.5	0.003	71	87.9	44.9–131.6	0.001	1.3	0.02–9.0	13.4	7.0–24.3	0.076
Healthy weight	424	14.7	13.8–16.0		4.7	3.0–7.4		422	64.4	33.1–119.3		5.8	3.7–8.9	14.9	11.1–19.7	
Overweight without obesity	205	15.0	13.8–16.2		4.5	2.4–8.5		206	87.4	41.4–155.4		5.8	3.2–10.2	22.2	16.0–29.8	
Obesity	197	14.5	13.3–16.2		12.0	7.5–18.6		199	95.3	44.8–185.8		3.6	1.7–7.2	24.3	17.9–32.1	

*n* = number of observations; CI = confidence interval; NO = no observations; y = years. * Anemia was defined as hemoglobin <13 g/dL for males aged ≥15 years and <12 g/dL for others [5]. † Serum ferritin concentration was adjusted by the regression correction approach using linear regression when the C-reactive protein concentration was >2 mg/L. A correction factor was obtained in this study. ‡ Ferritin assessment: <15 μg/L depleted iron stores; 15–200 μg/L adequate for men; 15–150 μg/L adequate for women; >200 μg/L risk of iron overload for men; >150 μg/L for women [3,5]. ‖ The *p*-value was obtained from the survey-weighted Theil–Sen median test. § The *p*-value was obtained from the survey-weighted chi-square tests. ¶ In international currency units, including purchasing power parity. ** BMI: For adolescents—underweight < 3rd percentile; healthy weight > 3rd percentile and <85th percentile; overweight > 85th percentile and <97th percentile; obese > 97th percentile [27]. For adults—underweight < 18.5 kg/m^2^; healthy weight 18.5–24.9 kg/m^2^; overweight 25.029.9 kg/m^2^; obese ≥ 30 kg/m^2^ [32]. For older adults—underweight < 23.0 kg/m^2^; healthy weight 23.0–27.9 kg/m^2^; overweight 28.0–29.9 kg/m^2^; obese ≥ 30 kg/m^2^ [33].

**Table 2:** Anemia vs. iron stores among residents of the urban area of São Paulo: 2015 Health Survey of São Paulo.

Characteristics	Depleted Iron Stores without Anemia *			Depleted Iron Stores and Anemia *,†			Anemia †			Anemia and Severe Risk of Iron Overload †,‡			Severe Risk of Iron Overload ‡			*p*-Value ‖
	*n*	%	95%CI	*n*	%	95%CI	*n*	%	95%CI	*n*	%	95%CI	*n*	%	95%CI	
Entire sample	40	4.0	2.8–5.7	11	1.1	0.5–2.1	42	4.3	3.1–6.0	10	1.3	0.6–2.6	127	17.6	14.7–20.87	-
Age groups																
Adolescents; 12–19 y	25	7.8	5.0–12.0	5	1.4	0.6–3.4	7	2.4	1.1–5.0	NO			7	2.3	1.0–4.7	<0.001
Adults; 20–59 y	13	3.9	2.2–6.9	2	0.7	0.2–3.1	12	4.0	2.2–7.0	4	1.6	0.6–4.2	70	25.3	20.3–31.0	
Older adults; ≥60 y	2	0.5	0.1–2.2	4	1.4	0.5–3.7	23	6.8	4.4–10.3	6	2.0	0.9–4.5	50	16.8	12.6–21.9	
Age groups and sex																
Male adolescents	2	1.3	0.3–5.3	NO			1	0.8	0.1–5.4	NO			7	4.6	2.2–9.5	<0.001
Female adolescents	23	14.0	8.9–21.4	5	2.8	1.2–6.6	6	3.9	1.7–8.7	NO			NO			
Male adults	NO			NO			2	1.4	0.4–5.6	4	3.0	1.1–7.9	70	47.6	39.3–56.1	<0.001
Female adults	13	8.4	4.8–14.3	2	1.5	0.3–5.4	10	6.8	3.6–12.6	NO			NO			
Male older adults	1	0.6	0.1–4.5	2	1.4	0.3–5.4	7	4.2	1.8–9.5	6	3.8	1.7–8.5	48	32.2	24.7–40.7	<0.001
Female older adults	1	0.4	0.0–2.9	2	1.4	0.3–5.5	16	9.5	5.8–15.4	NO			NO			
Leisure-time physical activity ^§^																
Insufficient physical activity	33	5.2	3.5–7.6	8	1.3	0.6–2.8	28	4.5	3.0–6.7	10	2.0	1.0–4.1	79	16.0	12.8–19.8	0.032
Sufficient physical activity	6	2.0	0.8–4.7	3	0.7	0.2–2.2	14	4.0	2.2–7.1	NO			48	20.4	15.2–26.8	
Body mass index classification **																
Underweight	NO			1	1.4	0.2–9.2	5	6.6	2.5–16.3	2	2.4	0.6–9.3	8	11.2	5.3–22.0	0.025
Healthy weight	20	4.4	2.6–7.2	6	1.4	0.5–3.6	16	2.9	1.7–5.0	2	0.4	0.1–1.8	52	14.5	10.7–19.3	
Overweight without obesity	13	5.5	3.0–10.0	1	0.3	0.0–2.0	8	3.2	1.5–6.8	2	1.0	0.2–4.4	33	21.2	15.2–28.8	
Obesity	6	2.7	1.2–6.2	3	1.2	0.4–3.8	13	7.7	4.2–13.4	4	3.0	1.0–8.6	34	21.4	15.4–29.0	

*n* = number of observations; % = prevalence; CI = confidence interval; NO = no observations; y = years. * Depleted iron stores were defined when serum ferritin was <15 μg/L [3,5]. † Anemia was defined when hemoglobin <13 g/dL for men aged ≥15 years and <12 g/dL for others [5]. ‡ Severe risk of iron overload was defined when ferritin was >150 µg/L in females and >200 µg/L in males [3,5,6]. Serum ferritin concentration was adjusted by the regression correction approach using linear regression when the C-reactive protein concentration was >2 mg/L. A correction factor was obtained in this study. ‖ The *p*-value was obtained from the survey-weighted chi-square tests. § Sufficient physical activity was considered when the individual performed moderate-intensity exercise for at least 30 min daily for 5 days per week or vigorous-intensity exercise for at least 20 min per day, three days per week [30,31]. ** For adolescents, underweight: BMI < 3rd percentile; healthy weight: BMI > 3rd percentile and <85th percentile; overweight: BMI > 85th percentile and <97th percentile; obese: BMI > 97th percentile [27]. For adults–underweight: BMI < 18.5 kg/m^2^; healthy weight: BMI 18.5–24.9 kg/m^2^; overweight: BMI 25.0–29.9 kg/m^2^; obese: BMI ≥ 30 kg/m^2^ [32]. For older adults–underweight: BMI < 23.0 kg/m^2^; healthy weight: BMI 23.0–27.9 kg/m^2^; overweight: BMI 28.0–29.9 kg/m^2^; obese: BMI ≥ 30 kg/m^2^ [33].

**Table 3:** Dietary and supplemental iron intake and the probability of inadequate dietary iron intake among residents of the urban area of São Paulo, according to sociodemographic and lifestyle factors: 2015 Health Survey of São Paulo.

Characteristics	*n*	Dietary Iron Intake (mg/d)			Dietary and Supplement Iron Intake (mg/d)			Probability of Inadequate Dietary Iron Intake *	
		Median	Interquartile Range	*p*-Value †	Median	Interquartile Range	*p*-Value †	%	95%CI
Entire sample	890	9.7	7.8–11.8	-	9.7	7.9–12.1	-	13.9	8.8–22.7
Age groups									
Adolescents; 12–19 y	287	10.3	8.5–13.3	<0.001	10.3	8.5–13.3	<0.001	18.4	9.3–38.3
Adults; 20–59 y	297	9.8	8.1–12.0		9.9	8.1–12.2		14.4	7.2–31.6
Older adults; ≥60 y	306	8.5	7.1–10.1		8.7	7.3–10.3		8.6	6.0–19.7
Age groups and sex									
Male adolescents	139	12.0	9.1–14.7	<0.001	12.0	9.1–14.8	<0.001	10.7	3.3–37.0
Female adolescents	148	9.8	8.1–11.4		9.8	8.2–11.4		25.8	11.9–60.6
Male adults	152	10.6	8.8–13.0	<0.001	10.9	8.8–13.6	<0.001	3.4	1.2–11.2
Female adults	145	8.8	7.1–10.6		8.9	7.2–10.6		36.4	12.9–61.9
Male older adults	151	9.2	7.7–10.6	<0.001	9.3	8.0–11.3	<0.001	8.8	3.4–25.1
Female older adults	155	7.8	6.9–9.5		8.0	7.0–9.7		8.4	3.5–24.6
Household head education									
<10 y	460	9.4	7.6–11.3	0.004	9.5	7.8–11.6	0.015	15.0	8.0–29.4
≥10 y	409	10.0	8.1–12.3		10.0	8.2–12.5		12.2	7.8–26.3
Leisure-time physical activity ^‡^									
Insufficient physical activity	589	9.3	7.6–11.2	0.003	9.4	7.6–11.5	0.002	15.7	9.3–27.6
Sufficient physical activity	299	10.0	8.3–12.2		10.0	8.4–12.6		10.7	4.7–28.6
Body mass index classification ^§^									
Underweight	71	9.7	6.9–12.0	0.008	9.9	7.7–14.3	0.001	11.4	3.3–43.5
Healthy weight	418	10.0	8.1–12.4		10.0	8.2–12.8		13.0	7.0–25.6
Overweight without obesity	202	9.4	7.6–11.3		9.4	7.6–11.4		16.6	7.4–41.0
Obesity	198	9.2	7.8–11.0		9.2	7.8–11.5		13.3	5.5–35.7

*n* = number of observations; CI = confidence interval; y = years. * Estimate average requirement for dietary iron: 13 years–5.9 mg/d for male and 5.7 mg/d for female; 14–18 years–7.7 mg/d for men and 7.9 mg/d para meninas; 19–50 years–6.0 mg/d for men and 8.1 mg/d for women; 51 years and more–6.0 mg/d for men and 5.0 mg/d for women [43]. † *p*-value obtained from the survey-weighted Theil–Sen median test. ‡ Sufficient physical activity was considered when the individual performed moderate-intensity exercise for at least 30 min daily for 5 days per week or vigorous-intensity exercise for at least 20 min per day, 3 days per week [30,31]. § For adolescents, underweight: BMI < 3rd percentile; healthy weight: BMI > 3rd percentile and <85th percentile; overweight: BMI > 85th percentile and <97th percentile; obese: BMI > 97th percentile [27]. For adults–underweight: BMI < 18.5 kg/m^2^; healthy weight: BMI 18.5–24.9 kg/m^2^; overweight: BMI 25.0–29.9 kg/m^2^; obese: BMI ≥ 30 kg/m^2^ [32]. For older adults–underweight: BMI < 23.0 kg/m^2^; healthy weight: BMI 23.0–27.9 kg/m^2^; overweight: BMI 28.0–29.9 kg/m^2^; obese: BMI ≥ 30 kg/m^2^ [33].

**Table 4:** Top ten food contributors to dietary iron intake among residents of the urban area of São Paulo: 2015 Health Survey of São Paulo.

Main Group	Subgroup	Category	Entire Sample			Adolescents; 12–19 y									Adults; 20–59 y									Older Adults; ≥60 y								
						Male			Female			Total			Male			Female			Total			Male			Female			Total		
			mg/d	SD	%C	mg/d	SD	%C	mg/d	SD	%C	mg/d	SD	%C	mg/d	SD	%C	mg/d	SD	%C	mg/d	SD	%C	mg/d	SD	%C	mg/d	SD	%C	mg/d	SD	%C
Grains	Breads, rolls, tortillas	Yeast breads	2.4	1.2	22.2	3.3	1.8	20.5	2.5	1.1	18.8	2.9	1.5	19.8	2.7	1.4	23.2	2.2	0.9	25.0	2.5	1.2	24.0	2.1	0.8	22.6	1.8	0.9	23.9	2.0	0.9	23.2
Protein foods	Plant-based protein foods	Beans, peas, and other legumes	1.4	0.9	14.4	1.6	0.7	13.4	1.3	0.7	12.6	1.5	0.7	13.1	1.7	1.0	17.7	1.1	0.8	10.6	1.5	1.0	14.8	1.4	1.1	18.8	1.0	0.8	12.1	1.2	1.0	15.7
Protein foods	Meats	Beef, excludes ground	2.0	1.4	9.0	2.4	1.5	7.5	1.9	1.8	9.1	2.2	1.7	8.2	2.3	1.6	10.8	1.6	1.2	7.6	2.0	1.5	9.5	1.6	1.0	10.0	1.7	0.9	8.9	1.7	0.9	9.5
Mixed dishes	Mixed dishes, grain-based	Turnovers and other grain-based items	2.0	1.6	4.1	2.1	1.4	5.4	1.8	1.5	4.8	2.0	1.4	5.1	2.4	2.0	3.5	1.9	1.4	5.5	2.1	1.7	4.3	1.6	1.2	2.0	1.3	1.2	3.3	1.5	1.2	2.6
Snacks and sweets	Sweet bakery products	Cookies and brownies	3.4	3.2	4.1	5.0	3.8	10.4	2.9	2.6	6.5	4.1	3.4	8.6	4.3	3.4	2.6	-	-	-	2.9	2.9	2.0	-	-	-	-	-	-	-	-	-
Mixed dishes	Mixed dishes, grain-based	Pasta mixed dishes	2.6	1.5	3.0	3.7	1.7	3.5	2.8	1.3	2.8	3.3	1.6	3.2	2.5	1.3	2.6	2.5	1.8	3.4	2.5	1.5	2.9	2.5	1.5	2.8	1.8	0.9	2.8	2.2	1.2	2.8
Snacks and sweets	Crackers	Saltine crackers	1.4	1.4	2.8	-	-	-	1.5	1.4	3.1	1.6	1.5	2.4	2.3	1.9	3.4	1.3	1.2	3.2	1.7	1.6	3.3	1.0	0.7	2.6	0.9	0.4	3.4	1.0	0.5	3.0
Protein foods	Meats	Ground beef and items made from ground beef	2.2	1.4	2.4	2.3	1.0	3.6	2.2	1.3	3.8	2.3	1.2	3.7	-	-	-	1.8	1.3	2.3	-	-	-	-	-	-	2.1	1.8	2.5	-	-	-
Grains	Breads, rolls, tortillas	Rolls and buns	2.1	1.4	2.4	2.5	1.4	2.1	2.0	1.0	2.6	-	-	-	2.4	1.8	2.3	2.0	1.3	4.5	2.2	1.5	3.2	-		-	-	-	-	-	-	-
Mixed dishes	Mixed dishes, pizza	Pizza	2.6	1.4	2.2	-	-	-	2.4	1.1	2.6	-	-	-	3.4	1.6	4.0	-	-	-	2.8	1.6	3.0	-		-	-	-	-	-	-	-
Grains	Cooked grains	Pasta, noodles, cooked grains	-	-	-	3.6	2.0	2.9	-	-	-	3.2	2.0	3.1	-	-	-	-	-	-	-	-	-	-		-	-	-	-	-	-	-
Sugars	Sugars	Jams, syrups, toppings	-	-	-	1.4	0.7	2.1	-	-	-	1.2	0.8	2.8	-	-	-	-	-	-	-	-	-	-		-	-	-	-	-	-	-
Protein foods	Poultry	Chicken, whole pieces	-	-	-	-	-	-	-	-	-	-	-	-	0.6	0.5	2.5	-	-	-	0.6	0.4	2.3	0.5	0.4	2.0	0.5	0.7	2.4	0.5	0.6	2.2
Snacks and sweets	Sweet bakery products	Cakes and pies	-	-	-	-	-	-	-	-	-	-	-	-	-	-	-	0.9	0.7	2.5	-	-	-	-	-	-	-	-	-	-	-	-
Mixed dishes	Mixed dishes, soups	Soups	-	-	-	-	-	-	-	-	-	-	-	-	-	-	-	1.6	1.8	2.3	-	-	-	-	-	-	1.7	2.0	3.3	1.7	1.9	2.6
Grains	Cereals	Grits and other cooked cereals	-	-	-	-	-	-	-	-	-	-	-	-	-	-	-	-	-	-	-	-	-	3.2	3.8	3.1	-	-	-	-	-	-
Grains	Ready-to-eat cereals	Ready-to-eat cereal, lower sugar (≤21.2 g/100 g)	-	-	-	-	-	-	-	-	-	-	-	-	-	-	-	-	-	-	-	-	-	12.2	18.6	2.7	8.6	19.0	5.4	9.8	17.8	4.0
Grains	Cooked grains	Rice	-	-	-	-	-	-	-	-	-	-	-	-	-	-	-	-	-	-	-	-	-	0.2	0.1	2.2	-	-	-	0.2	0.1	2.0
Total			-	-	66.6	-	-	71.4	-	-	66.7	-	-	70.0	-	-	72.6	-	-	66.9	-	-	69.3	-	-	68.8	-	-	68.0	-	-	67.6

mg/d and SD: mean and standard deviation of iron intake considering the number of times each food has been consumed; %C: percentage of contribution to dietary iron intake.

**Table 5:** Multiple linear regression analysis of hemoglobin and ferritin with iron/anemia blood biomarkers, sociodemographic and lifestyle factors in non-institutionalized residents of the urban area of São Paulo: 2015 Health Survey of São Paulo.

Factors	Hemoglobin (g/dL)		Ferritin (µg/L) *	
	β	95%CI	β	95%CI
Sex (ref. Men)				
Women	−1.17	−1.57; −0.77	−0.91	−1.04; −0.78
Age (ref. Adolescents; 12–19 years)				
Adults; 20–59 years	0.57	0.14; 0.99	0.80	0.67; 0.94
Older adults; ≥60 years	0.21	−0.32; 0.75	0.87	0.71; 1.03
Household head education (ref. < 10 years)				
≥10 years	-	-	−0.15	−0.31; 0.00
Family income *per capita* (ref. 1st tertile)				
2nd tertile	0.57	−0.05; 1.19	0.03	−0.16; 0.22
3rd tertile	0.81	0.18; 1.45	0.18	−0.02; 0.37
Leisure-time physical activity (ref. Insufficient physical activity)				
Sufficient physical activity	0.28	−0.46; 0.51	−0.08	−0.19; 0.04
Constant	14.96	14.55; 15.38	4.13	3.99; 4.27

Variables with a *p*-value ≥ 0.20 in the homoscedasticity test were not included in the models. * Serum ferritin concentration was transformed into a natural logarithm to obtain a normal distribution.

## Data Availability

Restrictions apply to the availability of these data. The data are not publicly available due to the fact that they contain information that could compromise the privacy of research participants. Interested parties should contact the corresponding author to discuss access permission without violating ethical commitments.

## References

[1] McLean E., Cogswell M., Egli I., Wojdyla D., de Benoist B. (2009). Worldwide prevalence of anaemia, WHO Vitamin and Mineral Nutrition Information System, 1993–2005. Public Health Nutr..

[2] Levi M., Simonetti M., Marconi E., Brignoli O., Cancian M., Masotti A., Pegoraro V., Heiman F., Cricelli C., Lapi F. (2019). Gender differences in determinants of iron-deficiency anemia: A population-based study conducted in four European countries. Ann. Hematol..

[3] World Health Organization (2001). Iron Deficiency Anaemia: Assessment, Prevention, and Control. A Guide for Programme Managers.

[4] World Health Organization (2007). Assessing the Iron Status of Populations: Including Literature Review.

[5] World Health Organization (2017). Nutritional Anaemias: Tools for Effective Prevention and Control.

[6] World Health Organization (2020). WHO Guideline on Use of Ferritin Concentrations to Assess Iron Status in Individuals and Populations.

[7] Petry N., Olofin I., Hurrell R.F., Boy E., Wirth J.P., Moursi M., Donahue Angel M., Rohner F. (2016). The Proportion of Anemia Associated with Iron Deficiency in Low, Medium, and High Human Development Index Countries: A Systematic Analysis of National Surveys. Nutrients.

[8] Kaidar-Person O., Person B., Szomstein S., Rosenthal R.J. (2008). Nutritional deficiencies in morbidly obese patients: A new form of malnutrition? Part B: Minerals. Obes. Surg..

[9] Batista Filho M., Souza A.I., Miglioli T.C., Santos M.C. (2008). Anemia and obesity: A paradox of the nutritional transition in Brazil. Cad. Saude Publica.

[10] Wells J.C., Sawaya A.L., Wibaek R., Mwangome M., Poullas M.S., Yajnik C.S., Demaio A. (2020). The double burden of malnutrition: Aetiological pathways and consequences for health. Lancet.

[11] Becker C., Orozco M., Solomons N.W., Schümann K. (2015). Iron metabolism in obesity: How interaction between homoeostatic mechanisms can interfere with their original purpose. Part I: Underlying homoeostatic mechanisms of energy storage and iron metabolisms and their interaction. J. Trace Elem. Med. Biol..

[12] Strenghtening Partnerships, Results, and Innovations in Nutrition Globally (SPRING) (2017). Changing the Way We Think about Micronutrient Assessment and Anemia Programming. Findings from the Biomarkers Reflecting Inflammation and Nutritional Determinants of Anemia (BRINDA) Project.

[13] De Benoist B., McLean E., Egli I., Cogswell M. (2008). Worldwide Prevalence of Anaemia 1993–2005. WHO Global Database on Anaemia.

[14] Eckhardt C.L., Torheim L.E., Monterrubio E., Barquera S., Ruel M.T. (2008). The overlap of overweight and anaemia among women in three countries undergoing the nutrition transition. Eur. J. Clin. Nutr..

[15] Traissac P., Montenegro R., El Ati J., Gartner A., Ben Gharbia H., Bour A., Delpeuch F. (2020). In a nutrition transition context in North Africa, is the co-occurrence of excess adiposity and iron deficiency or anemia independent, aggravating or protective?. Curr. Dev. Nutr..

[16] Brasil. Ministério da Saúde. Agência Nacional de Vigilância Sanitária (2002). Resolução de Diretoria Colegiada—RDC N° 344, de 13 de Dezembro de 2002.

[17] Brasil. Ministério da Saúde. Gabinete do Ministro (2009). Portaria n° 1793, de 11 de Agosto de 2009. Institui a Comissão Interinstitucional para Implementação, Acompanhamento e Monitoramento das Ações de Fortificação de Farinhas de Trigo, de Milho e de seus Subprodutos.

[18] Buzzo M., Carvalho M., Tiglea P., Arauz L., Arakaki E., Matsuzaki R. (2012). Monitoring the wheat and corn flours enriched with iron. Rev. Do Inst. Adolfo Lutz.

[19] Kira C.S., Buzzo M.L., Carvalho M.d.F.H., Duran M.C., Sakuma A.M. (2006). Evaluation of iron content in fortified wheat flour, São Paulo, Brazil. Rev. Do Inst. Adolfo Lutz.

[20] Vieira D.A.d.S., Steluti J., Verly E., Marchioni D.M., Fisberg R.M. (2017). Brazilians’ experiences with iron fortification: Evidence of effectiveness for reducing inadequate iron intakes with fortified flour policy. Public Health Nutr..

[21] Dos Santos Q., Nilson E.A., Verly Junior E., Sichieri R. (2015). An evaluation of the effectiveness of the flour iron fortification programme in Brazil. Public Health Nutr..

[22] Assunção M.C., Santos I.S., Barros A.J., Gigante D.P., Victora C.G. (2012). Flour fortification with iron has no impact on anaemia in urban Brazilian children. Public Health Nutr..

[23] Szarfarc S.C. (2010). Public policies to control iron deficiency in Brazil. Rev. Bras. Hematol. Hemoter..

[24] Fisberg R.M., Sales C.H., Fontanelli M.M., Pereira J.L., Alves M.C.G.P., Escuder M.M.L., César C.L.G., Goldbaum M. (2018). 2015 Health Survey of São Paulo with Focus in Nutrition: Rationale, Design, and Procedures. Nutrients.

[25] Alves M.C.G.P., Escuder M.M.L., Goldbaum M., Barros M.B.A., Fisberg R.M., Cesar C.L.G. (2018). Sampling plan in health surveys, city of São Paulo, Brazil, 2015. Rev. Saude Publica.

[26] Brasil. Presidência da República. Casa Civil. Subchefia para Assuntos Jurídicos (1990). Lei n° 8069, de 13 de Julho de 1990. Dispõe Sobre o Estatuto da Criança e do Adolescente e dá Outras Providências.

[27] De Onis M., Onyango A.W., Borghi E., Siyam A., Nishida C., Siekmann J. (2007). Development of a WHO growth reference for school-aged children and adolescents. Bull. World Health Organ..

[28] Brasil. Presidência da República. Casa Civil. Subchefia para Assuntos Jurídicos (2003). Lei n° 10741, de 1° de Outubro de 2003. Dispõe Sobre o Estatuto do Idoso e dá Outras Providências.

[29] Craig C.L., Marshall A.L., Sjostrom M., Bauman A.E., Booth M.L., Ainsworth B.E., Pratt M., Ekelund U., Yngve A., Sallis J.F. (2003). International physical activity questionnaire: 12-country reliability and validity. Med. Sci. Sports Exerc..

[30] Nelson M.E., Rejeski W.J., Blair S.N., Duncan P.W., Judge J.O., King A.C., Macera C.A., Castaneda-Sceppa C. (2007). Physical activity and public health in older adults: Recommendation from the American College of Sports Medicine and the American Heart Association. Med. Sci. Sports Exerc..

[31] Haskell W.L., Lee I.M., Pate R.R., Powell K.E., Blair S.N., Franklin B.A., Macera C.A., Heath G.W., Thompson P.D., Bauman A. (2007). Physical activity and public health: Updated recommendation for adults from the American College of Sports Medicine and the American Heart Association. Med. Sci. Sports Exerc..

[32] World Health Organization (2000). Obesity: Preventing and Managing the Global Epidemic. Report of a WHO Consultation (WHO Technical Report Series 894).

[33] Lebrão M.L., Duarte Y.A.d.O. (2003). SABE-Saúde, Bem-Estar e Envelhecimento-O Projeto Sabe no município de São Paulo: Uma Abordagem Inicial.

[34] Yamanishi H., Iyama S., Yamaguchi Y., Kanakura Y., Iwatani Y. (2003). Total iron-binding capacity calculated from serum transferrin concentration or serum iron concentration and unsaturated iron-binding capacity. Clin. Chem..

[35] Gartner A., Berger J., Bour A., El Ati J., Traissac P., Landais E., El Kabbaj S., Delpeuch F. (2013). Assessment of iron deficiency in the context of the obesity epidemic: Importance of correcting serum ferritin concentrations for inflammation. Am. J. Clin. Nutr..

[36] Namaste S.M., Aaron G.J., Varadhan R., Peerson J.M., Suchdev P.S., Group B.W. (2017). Methodologic approach for the Biomarkers Reflecting Inflammation and Nutritional Determinants of Anemia (BRINDA) project. Am. J. Clin. Nutr..

[37] Grotto H.Z.W. (2010). Laboratory diagnosis of iron deficiency anemia. Rev. Bras. Hematolgia Hemoter..

[38] Raper N., Perloff B., Ingwersen L., Steinfeldt L., Anand J. (2004). An overview of USDA’s Dietary Intake Data System. J. Food Compos. Anal..

[39] Moshfegh A.J., Rhodes D.G., Baer D.J., Murayi T., Clemens J.C., Rumpler W.V., Paul D.R., Sebastian R.S., Kuczynski K.J., Ingwersen L.A. (2008). The US Department of Agriculture Automated Multiple-Pass Method reduces bias in the collection of energy intakes. Am. J. Clin. Nutr..

[40] Núcleo de Estudos e Pesquisas em Alimentação. Universidade Estadual de Campinas (2011). Universidade Estadual de Campinas. Tabela Brasileira de Composição de Alimentos—TACO.

[41] Harttig U., Haubrock J., Knüppel S., Boeing H., Consortium E. (2011). The MSM program: Web-based statistics package for estimating usual dietary intake using the Multiple Source Method. Eur. J. Clin. Nutr..

[42] National Research Council (US) (1986). Subcommitte on Criteria for Dietary Evaluation. Nutrient Adequacy: Assessment Using Food Consumption Surveys.

[43] Institute of Medicine (2001). Dietary Reference Intakes for Vitamin A, Vitamin K, Arsenic, Boron, Chromium, Copper, Iodine, Iron, Manganese, Molybdenum, Nickel, Silicon, Vanadium, and Zinc.

[44] National Research Council (US) List of WWEIA Food Categories. https://www.ars.usda.gov/northeast-area/beltsville-md-bhnrc/beltsville-human-nutrition-research-center/food-surveys-research-group/docs/dmr-food-categories/.

[45] Fisberg R.M., Leme A.C.B., Previdelli Á., Mello A.V., Arroyo A.M., Sales C.H., Gómez G., Kovalskys I., Herrera-Cuenca M., Sanabria L.Y.C. (2021). Contribution of food groups to energy, grams and nutrients-to-limit: The Latin American Study of Nutrition and Health/Estudio Latino Americano de Nutrición y Salud (ELANS). Public Health Nutr..

[46] Stata Corp LP (2013). Stata Survey Data Reference Manual: Release 13.

[47] Newson R. (2006). Confidence intervals for rank statistics: Percentile slopes, differences, and ratios. Stata J..

[48] D’Agostino R.B., Belanger A., D’Agostino R.B. (1990). A suggestion for using powerful and informative tests of normality. Am. Stat..

[49] Royston P. (1992). Comment on sg3.4 and an improved D’Agostino test. Stata Tech. Bull..

[50] De Andrade Cairo R.C., Rodrigues Silva L., Carneiro Bustani N., Ferreira Marques C.D. (2014). Iron deficiency anemia in adolescents; a literature review. Nutr. Hosp..

[51] Brasil. Minstério da Saúde. Agência Nacional de Vigilância Sanitária. Diretoria Colegiada (2017). Resolução RDC n° 150 de 13 de Abril de 2017. Requisitos Para o Enriquecimento de Farinhas de Trigo e de Milho Com Ferro e Ácido Fólico.

[52] Brasil. Ministério da Economia. Instituto Brasileiro de Geografia e Estatística. Diretoria de Pesquisas. Coordenação de Trabalho e Rendimentos (2020). Pesquisa de Orçamentos Familiares 2017–2018: Avaliação da Disponibilidade Domiciliar de Alimentos no Brasil.

[53] Bouri S., Martin J. (2018). Investigation of iron deficiency anaemia. Clin. Med..

[54] Jones A.D., Hayter A.K., Baker C.P., Prabhakaran P., Gupta V., Kulkarni B., Smith G.D., Ben-Shlomo Y., Krishna K.V., Kumar P.U. (2016). The co-occurrence of awasnemia and cardiometabolic disease risk demonstrates sex-specific sociodemographic patterning in an urbanizing rural region of southern India. Eur. J. Clin. Nutr..

[55] Wiciński M., Liczner G., Cadelski K., Kołnierzak T., Nowaczewska M., Malinowski B. (2020). Anemia of Chronic Diseases: Wider Diagnostics-Better Treatment?. Nutrients.

[56] Machado Í.E., Malta D.C., Bacal N.S., Rosenfeld L.G.M. (2019). Prevalence of anemia in Brazilian adults and elderly. Rev. Bras. Epidemiol..

[57] Santos M.T.L.D., Costa K.M.M., Bezerra I.M.P., Santos E.F.S., Szarfarc S.C., Rocha Pereira M.J.F.D., Abreu L.C., Venancio D.P. (2020). Anemia and iron deficiency in primigent parturients in a municipality of Brazilian west Amazon. Medicine.

[58] Ferreira H.d.S., Bezerra M.K.d.A., de Assunção M.L., de Menezes R.C.E. (2016). Prevalence of and factors associated with anemia in school children from Maceió, northeastern Brazil. BMC Public Health.

[59] Lício J.S.A., Fávaro T.R., Chaves C.R.M.d.M. (2016). Anemia in indigenous women and children in Brazil: A systematic review. Ciência Saúde Coletiva.

[60] Vieira R.C.d.S., do Livramento A.R.S., Calheiros M.S.C., Ferreira C.M.X., dos Santos T.R., de Assunção M.L., Ferreira H.d.S. (2017). Prevalence and temporal trend (2005–2015) of anemia among children in Northeast Brazil. Public Health Nutr..

[61] World Health Organization (2011). Guideline: Intermittent Iron and Folic Acid Supplementation in Menstruating Women.

[62] Fisberg M., Tosatti A.M. (2011). Enrichment of iron and folic acid: The real need and the dangers of this initiative. Rev. Bras. Hematol. Hemoter..

[63] Martins J.M. (2011). Considerations on the food fortification policy in Brazil. Rev. Bras. Hematol. Hemoter..

[64] Cançado R.D., Chiattone C.S. (2010). Current approach to hereditary hemochromatosis. Rev. Bras. Hematol. Hemoter..

[65] Mainous A.G., Tanner R.J., Coates T.D., Baker R. (2014). Prediabetes, elevated iron and all-cause mortality: A cohort study. BMJ Open.

[66] Vieira D.A.d.S., Sales C.H., Cesar C.L.G., Marchioni D.M., Fisberg R.M. (2018). Influence of Haem, Non-Haem, and Total Iron Intake on Metabolic Syndrome and Its Components: A Population-Based Study. Nutrients.

[67] Lönnerdal B. (2017). Excess iron intake as a factor in growth, infections, and development of infants and young children. Am. J. Clin. Nutr..

[68] Paganini D., Zimmermann M.B. (2017). The effects of iron fortification and supplementation on the gut microbiome and diarrhea in infants and children: A review. Am. J. Clin. Nutr..

[69] Ng S.W., Norwitz S.G., Norwitz E.R. (2019). The Impact of Iron Overload and Ferroptosis on Reproductive Disorders in Humans: Implications for Preeclampsia. Int. J. Mol. Sci..

[70] Swanson C.A. (2003). Iron intake and regulation: Implications for iron deficiency and iron overload. Alcohol.

[71] Brissot P., Troadec M.B., Loréal O., Brissot E. (2019). Pathophysiology and classification of iron overload diseases; update 2018. Transfus. Clin. Biol..

[72] Basuli D., Stevens R.G., Torti F.M., Torti S.V. (2014). Epidemiological associations between iron and cardiovascular disease and diabetes. Front. Pharm..

[73] Zhang W., Iso H., Ohira T., Date O.C., Tanabe N., Kikuchi S., Tamakoshi A. (2012). Associations of dietary iron intake with mortality from cardiovascular disease: The JACC study. J. Epidemiol..

[74] Skikne B.S., Punnonen K., Caldron P.H., Bennett M.T., Rehu M., Gasior G.H., Chamberlin J.S., Sullivan L.A., Bray K.R., Southwick P.C. (2011). Improved differential diagnosis of anemia of chronic disease and iron deficiency anemia: A prospective multicenter evaluation of soluble transferrin receptor and the sTfR/log ferritin index. Am. J. Hematol..

[75] Yanoff L.B., Menzie C.M., Denkinger B., Sebring N.G., McHugh T., Remaley A.T., Yanovski J.A. (2007). Inflammation and iron deficiency in the hypoferremia of obesity. Int. J. Obes..

[76] Cepeda-Lopez A.C., Aeberli I., Zimmermann M.B. (2010). Does obesity increase risk for iron deficiency? A review of the literature and the potential mechanisms. Int. J. Vitam. Nutr. Res..

[77] Cepeda-Lopez A.C., Osendarp S.J., Melse-Boonstra A., Aeberli I., Gonzalez-Salazar F., Feskens E., Villalpando S., Zimmermann M.B. (2011). Sharply higher rates of iron deficiency in obese Mexican women and children are predicted by obesity-related inflammation rather than by differences in dietary iron intake. Am. J. Clin. Nutr..

[78] Lecube A., Hernández C., Pelegrí D., Simó R. (2008). Factors accounting for high ferritin levels in obesity. Int. J. Obes..

[79] González-Domínguez Á., Visiedo-García F.M., Domínguez-Riscart J., González-Domínguez R., Mateos R.M., Lechuga-Sancho A.M. (2020). Iron metabolism in obesity and metabolic syndrome. Int. J. Mol. Sci..

[80] Guralnik J.M., Eisenstaedt R.S., Ferrucci L., Klein H.G., Woodman R.C. (2004). Prevalence of anemia in persons 65 years and older in the United States: Evidence for a high rate of unexplained anemia. Blood.

[81] Tschopp J. (2011). Mitochondria: Sovereign of inflammation?. Eur. J. Immunol..

[82] Jimenez K.M., Gasche C. (2019). Management of Iron Deficiency Anaemia in Inflammatory Bowel Disease. Acta Haematol..

[83] Salas G.G., Sanabria A.R., Oreamuno A.S., Chinnock A., Previdelli A.N., Sales C.H., Quesada D.Q. (2019). Prevalencia de ingesta inadecuada de micronutrientes en la población urbana de Costa Rica. Arch. Latinoam. Nutr..

[84] Rhodes E.C., Suchdev P.S., Narayan K.M.V., Cunningham S., Weber M.B., Tripp K., Mapango C., Ramakrishnan U., Hennink M., Williams A.M. (2020). The co-occurrence of overweight and micronutrient deficiencies or anemia among women of reprodutive age in Malawi. J. Nutr..

[85] De Carvalho A.M., César C.L., Fisberg R.M., Marchioni D.M. (2013). Excessive meat consumption in Brazil: Diet quality and environmental impacts. Public Health Nutr..

[86] Low M.S., Speedy J., Styles C.E., De-Regil L.M., Pasricha S.R. (2016). Daily iron supplementation for improving anaemia, iron status and health in menstruating women. Cochrane Database Syst. Rev..

